# Can femoral neck-shaft angle predict timing of contralateral second hip fracture? A 7-year retrospective cohort study at a tertiary referral centre for trauma

**DOI:** 10.1007/s12306-025-00908-7

**Published:** 2025-07-15

**Authors:** Ezanul Harriz Abd Wahab, Colum Downey, Ben Murphy, Sophie Lawlor, Patrick O’kelly, Conor Shortt, John F. Quinlan

**Affiliations:** 1 Tallaght University Hospital, Dublin, Ireland; 2 Royal College of Surgeons in Ireland, Dublin, Ireland; 3 Trinity College Dublin, Dublin, Ireland; 4 Beaumont Hospital, Dublin, Ireland

**Keywords:** Hip fracture, Second hip fracture, Neck-shaft angle, Fracture liaison

## Abstract

**Introduction:**

Previously published literature from our institution found that patients with a fragility hip fracture were estimated to have a 4–10% risk of sustaining a second contralateral hip fracture. A follow-up, multi-centre study found that 1 in 11 (9.1%) patients sustained a contralateral hip fracture within three years of index hip fracture. Previous studies have examined the anatomic geometry of the hip joint as a risk factor for hip fractures. Our study aimed to establish a relationship between the neck-shaft angle (NSA) of the contralateral hip in patients who had already suffered a hip fragility fracture in terms of timing to second hip fracture.

**Methods:**

A 7-year, single-institution, retrospective cohort study of patients that presented with a second contralateral fragility hip fracture from 2013 to 2019 were reviewed. Inclusion criteria were all patients 60 years old and above who suffered a second contralateral hip fracture. Exclusion criteria were all patients who were aged less than 60 years old, high-energy injuries or those who suffered peri-prosthetic fractures. The NSA was calculated by measuring the intersection of the femoral neck axis and the femoral shaft axis of the hip. Age, gender, surgery type and American Society of Anaesthesiologists Physical Status Classification (ASA) score were also examined.

**Results:**

Ninety-four patients were suitable for analysis. NSA ranged from 113 to 146.5 degrees with an average of 130.2 degrees. Female patients had an average NSA of 129.7 degrees compared to 131.3 degrees in male patients. Average time to second hip fracture was 3.5 years, ranging from 0.08 years (29 days) to 20 years (7326 days). There was a 2.3:1 ratio of female-to-male presentations. Patient age ranged from 60 to 100 years old. The largest age group included patients aged 80–89 years, with 38 patients (28 females and 10 males). Correlation analysis performed showed no statistical significance between NSA and timing of second contralateral hip fracture with a *p* value of 0.235. There was an association between fracture type, specifically intracapsular hip fractures, and time to second hip fracture, but this was not statistically significant (*p* value 0.052).

**Conclusion:**

There is no statistically significant association between femoral NSA and time to second fragility hip fracture. As we have excluded NSA as an independent risk factor, further studies may now be carried out to look for other potential predictors of timing to second hip fracture.

## Introduction

Fragility hip fractures are one of the most common orthopaedic conditions necessitating presentation to and treatment in acute hospitals [1–4]. Globally, approximately 2 million hip fractures occur annually, and this is predicted to increase by three- to fourfold by 2050 [2]. Recent figures from the National Office of Clinical Audit (NOCA) in our country recorded 3,701 hip fractures in patients aged 60 and over, accounting for 99% of all hip fractures nationally [3]. These injuries remain a significant cause of morbidity and mortality. They are associated with a 30-day mortality rate of 5.7–6.5% and 1-year mortality rate of 22% [1, 2]. In the country where our study was completed, hip fractures are estimated to cost approximately €54 million per year with a projected cost increase to approximately €162 million per year by 2046 [2].

The British Orthopaedic Association (BOA) ‘The Care of Patients with Fragility Fracture’ guidelines [4] and BOA Standards for Trauma and Orthopaedics (BOAST) fragility hip fracture guidelines are an attempt to address hip fracture care in a safe and efficient manner [5]. In our country, a national hip fracture database was created to capture accurate short- and long-term data relating to the care of fragility hip fractures in our country. This database is under the clinical governance of NOCA, who then publish an annual report on the care of fragility hip fractures [3].

A previous study from our institution found that patients with a fragility hip fracture were estimated to have a 4–10% risk of sustaining a second contralateral hip fracture [1, 2, 6]. A follow-up, higher-powered, multi-centre study involving our institution and 5 others within the region found that 1 in 11 (9.1%) patients sustained a contralateral hip fracture within three years of index hip fracture [1, 2].

Risk factors for a contralateral second hip fracture have been identified in the literature and include: dementia, Parkinson’s disease, audio-visual impairment and older age at time of index fracture [6–8]. Previous studies have examined the anatomic geometry of the hip joint as a risk factor for hip fractures [9–11]. Hip axis length and neck-shaft angle (NSA) were independent parameters that predicted hip fractures [9, 12, 13]. In this study, we sought to establish a relationship between femoral NSA and second hip fracture, given our previous work on the contralateral hip fracture cohort. Given the morbidity and mortality associated with hip fractures, as well as their financial implications, we hoped to develop a clinical tool that could potentially predict, and thus prevent, a second hip fracture from occurring.

Thus, our study aimed to establish a relationship between the NSA of the contralateral hip in patients who had already suffered a hip fragility fracture. A secondary aim was to examine whether this relationship was an independent risk factor that could predict timing of second hip fracture.

## Patients and methods

A 7-year, single-institution, retrospective cohort study of patients that presented with a second contralateral fragility hip fracture from 2013 to 2019 were reviewed. The data were obtained from the office of the hip fracture database audit coordinator, who collects the data on behalf of the national office of clinical audit. Inclusion criteria were all patients 60 years old and above who suffered a second contralateral hip fracture. Exclusion criteria were all patients who were aged less than 60 years old, high-energy injuries or those who suffered peri-prosthetic fractures.

**Fig. 1:**
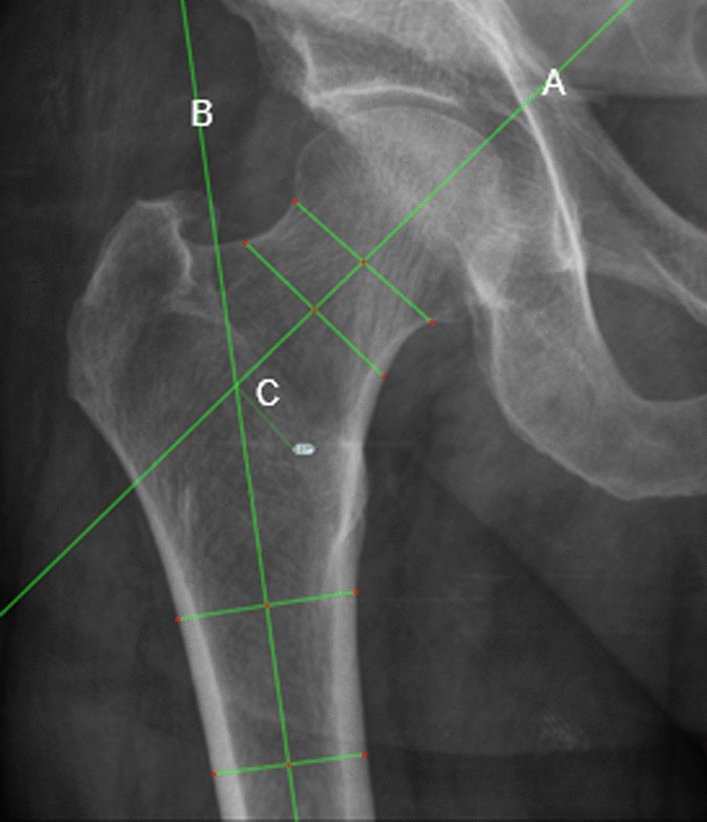
NSA measurements with TraumaCAD software. Line A is femoral neck axis; Line B is femoral shaft axis and C is intersection of line A and B, which is the femoral neck-shaft angle

Plain film anteroposterior (AP) pelvis radiographs were reviewed on the National Integrated Medical Imaging System (NIMIS). NSA of the contralateral hip prior to second hip fracture was measured using a standardized method with the NIMIS Trauma CAD software. We replicated a similar measuring technique described by Rippstein and Muller [14, 15]. The NSA was calculated by measuring the intersection of the femoral neck axis and the femoral shaft axis of the hip. The femoral neck axis is determined by locating the centre of the femoral head with a circular template. A line is drawn connecting the points where the circle intersects the medial and lateral border of the femoral neck. A line perpendicular to that line through the centre of the femoral head represents the femoral neck axis. The femoral shaft axis is drawn midway between the lateral and medial borders of the femoral shaft (Fig. 1). Measurements were taken independently by two senior specialist registrars in trauma and orthopaedic surgery (CD, BM), and an average of both authors’ NSA was calculated. Other categorical data were also collected: age, gender, type of surgical intervention and American Society of Anesthesiologists Physical Status Classification (ASA) score. Statistical analysis was performed with Stata SE (Version 13, College Station, Texas) statistical software, with a significance level of *p* value < 0.05 agreed upon.

## Results

After applying our exclusion criteria, 99 patients were identified as having sustained a contralateral second hip fracture at our institution between 2013 and 2019. Ninety-four patients were suitable for contralateral NSA analysis. Five patients were excluded due to poor radiograph quality, and thus, accurate NSA analysis could not be completed. Subgroup analysis was performed on all 99 patients with regard to age, gender, type of surgical procedure received and ASA score.

**Fig. 2:**
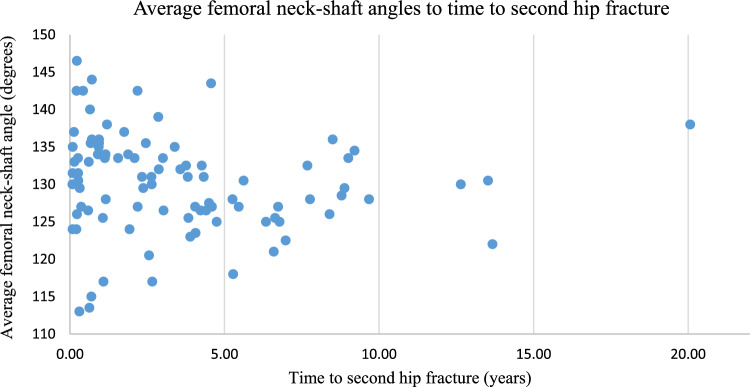
Distribution of average femoral neck-shaft angles compared to time to second hip fracture

NSA ranged from 113 to 146.5 degrees with an average of 130.2 degrees. Female patients had an average NSA of 129.7 degrees compared to 131.3 degrees in male patients. Average time to second hip fracture was 3.5 years, ranging from 0.08 years (29 days) to 20 years (7326 days). Fifty-six percent of second hip fractures occurred within 3 years of index fracture (Fig. 2).

In terms of hip fracture classification, there were 49 patients (52.1%) with intracapsular hip fractures, 43 patients (45.7%) with intertrochanteric hip fractures and 2 patients (2.1%) with subtrochanteric femur fractures.

There was a 2.3:1 ratio of female-to-male presentations with 69 female patients and 30 male patients. Patient age ranged from 60 to 100 years old. The largest age group included patients aged 80–89 years, with 38 patients (28 females and 10 males). This was followed by the 70–79 years group with 31 patients, the 90–99 years group with 20 patients, the 60–69 years group with 9 patients and 1 female patient aged 100 years old (Table 1).

**Table 1:** Distribution of age and gender of patients

Age (years)	Number (n)	Female	Male	Ratio (female: male)
60–69	9	3	6	0.5
70–79	31	21	10	2.1
80–89	38	28	10	2.8
90–99	20	16	4	4.0
> 99	1	1	0	1
Total	99	69	30	2.3

Looking at operative procedures, 42.4% of patients underwent hemiarthroplasty, 27.2% had intramedullary nail fixation, 23.2% had dynamic hip screw fixation, 5% underwent a total hip replacement and 1% of patients underwent screw fixation alone or were treated non-operatively (Table 2).

**Table 2:** Frequency and type of surgery

Type of surgery	Frequency	Percentage (%)	Cumulative
Hip hemiarthroplasty	42	42.42	42.42
Intramedullary nail fixation	27	27.27	69.69
Dynamic hip screw fixation	23	23.24	92.93
Total hip replacement	5	5.05	97.98
Screw fixation	1	1.01	98.99
Non-operative	1	1.01	100
Total	99	100	

An ASA score of 3 is defined as severe systemic disease that limits activity and was recorded in 61.6% of the hip fractures. An ASA score of 2, mild disease that does not limit activity, was found in 17.1% of patients. ASA 4, severe systemic disease that is a constant threat to life, and ASA 1, which corresponds to a normal, healthy patient, accounted for 9% and 1% of patients, respectively. No ASA score was documented in 11.1% of patients (Table 3).

**Table 3:** ASA grade of patients

ASA Grade	Frequency		Percentage (%)	Cumulative
1	1		1.01	1.01
2	17		17.17	18.18
3	61		61.62	79.80
4	9		9.09	88.89
Not documented	11		11.11	100.00
Total		99	100	

Correlation analysis performed showed no statistical significance between NSA and timing of second contralateral hip fracture with *p* value of 0.235. There was an association between fracture type, specifically intracapsular hip fractures, and time to second hip fracture. However, this was not statistically significant (*p* value 0.052). Similarly, no statistical significance was identified when examining other patient variables and timing to second hip fracture (Table 4).

**Table 4:** Correlation of variables to timing to second hip fracture

Variable	HR	95% Conf. Int	*p* Value
NSA (average)	1.023	0.985–1.063	0.235
Age	0.989	0.963–1.015	0.395
Gender	0.691	0.403–1.185	0.179
Fracture type	1.545	0.997–2.395	0.052

## Discussion

Our study demonstrated that femoral NSA is not a statistically significant independent risk factor in predicting time to contralateral second hip fracture. That said, there are some findings from our study that are in line with previously published research and other findings, which may be used to inform future research in this area. There are conflicting results reported previously with regard to the use of NSA as a risk factor for hip fracture. Gnudi [9] reported that hip fracture incidence is higher in females with wide NSA, and the combination of wide NSA and low bone mineral density confers the highest hip fracture incidence. A study by Ha et al. [10] also examined hip structural analysis and found that NSA, along with hip axis length, width of the intertrochanteric region and femoral shaft and bone mineral density are useful in predicting hip fracture. Tuck [16] reported that the NSA alone cannot accurately determine the risk of hip fracture in men based on their study with DEXA data. Ripamonti [11] also reported that NSA is associated with hip fracture risk in males but is not independent of femoral neck bone mineral density. All these studies share the common denominator of discussions regarding bone mineral density. Given what we know about the association between low bone mineral density and fragility fractures, it may be that NSA alone is not a significant risk factor in predicting time to contralateral hip fracture. Our study supports these findings as we examined NSA in isolation, further adding to the body of evidence.

Sheikh [17] has reported that in the UK population, patients with the highest risk of a second hip fractures were those with dementia, acute inpatient chest infection, urinary tract infection and multiple co-morbidities, as measured by the Charlson score. This score predicts 10-year survival in patients with multiple co-morbidities. Sawalha [8] also reported that patients more likely to suffer a second hip fracture were those who were institutionalized, female, older age and have lower mobility and mental test scores. This is corroborated by our study’s findings. Patients were more likely to be female (2:1). The majority of second hip fracture patients in this study (*n* = 89) were aged 70 and above, in line with what we know about older age as a risk factor. Although we did not find a statistically significant association between NSA and contralateral hip fracture, our study perhaps highlighted a need to further focus efforts on previously well-described risk factors such as being female and being of an older age. Knowing that these patients are more vulnerable may help to inform future clinical decision-making e.g. closer outpatient follow-up for female patients or those patients over 80 years of age, for instance.

The ASA score allows anaesthetists, and surgeons, by extension, to assess the pre-operative status of a patient. It will help identify those who are more at risk from a surgical procedure. Hip fracture patients are a particularly vulnerable cohort who are at risk of mortality from both operative and non-operative measures. Although no clear association between NSA and timing to second hip fracture was found, the association between a higher ASA score and this second hip fracture cohort is clear. 71.6% of patients were ASA 3 or above. This finding may prompt further research studies to investigate the relationship between second hip fracture and higher ASA scores. In terms of clinical practice, an awareness of this association between increasing ASA score and a contralateral second hip fracture may lead to more intensive preventive strategies in this cohort.

Out study has helped to add to the body of evidence that causes of second hip fractures are multifactorial and are not independently linked to any specific risk factors. Identification of these risk factors is key in improving the care and outcomes of patients. The International Osteoporosis Foundation (IOF) specifically endorses a multidisciplinary care model, recommending a dedicated coordinator acting as the liaison between both patient and the multidisciplinary team with their “Capturing the Fracture’ initiative [1, 2, 18]. The Fracture Liaison Service (FLS) is a multidisciplinary team that is designed to ensure improved outcomes in these patients and well as preventing fragility hip fractures. In the UK, McLellan et al. [19] have shown that with the provision of an FLS, 18 hip fractures are prevented in every 1000 fragility fracture patients with a cost saving of £21,000 to the exchequer.

NSA ranged from 113 to 146.5 degrees with an average of 130.2 degrees in our patient. It was hypothesized by one of the authors (CD) that the NSA in those fractured for a second time may have had more natural varus in their hips. An average of 130.2 degrees was not in agreement with this hypothesis as that is near normal for a native NSA. The majority of second hip fractures occurred within the first 5 years (Fig. 2). However, the spread of NSA within this timeframe made it too difficult to derive any meaningful conclusion. These fractures were concentrated in the 125 degrees to 130-degree range, which again disproves the theory that a more varus hip is more prone to fracture early. This finding is useful as it will allow future research efforts to focus on other potential non-modifiable risk factors other than NSA, given the negative findings of our study. Subgroup analysis of fracture type, specifically looking at intracapsular fractures, showed some association, but this was not statistically significant.

Our study has some limitations. It is a retrospective cohort study without any randomization. Our measuring technique relies on researcher familiarity with the measurement software and rotation of the femur on the plain film radiographs. Efforts to improve accuracy of NSA measurements were taken with repeated measurements made by each author; however, inter-observer variation should be acknowledged. The degree of rotation of the femur on radiographs was not standardized.

A strength of our study was that this was a comprehensive review of all second hip fractures in a busy trauma unit over a prolonged period. Although we found no association between NSA and timing to second hip fracture, our data collection and analysis uncovered important information regarding these second hip fracture patients. This may be used to inform future research and allows us to identify trends in second hip fracture incidence. The corroboration of female gender and advanced age as risk factors for second hip fracture is an important finding. These are non-modifiable risk factors and thus difficult to control for. This finding should encourage future research into potential modifiable risk factors to prevent second hip fractures in this vulnerable patient cohort. The exclusion of NSA as an independent predictor of timing to second hip fracture will allow others in the field to focus their future research on other variables. This will serve to benefit these vulnerable patients in the long run.

## Conclusion

There is no statistically significant association between femoral NSA and second fragility hip fracture. The NSA of most of the patients in the study was in the average range. Our study is in line with others in the literature in terms of risk factors for second hip fracture such as older age and gender. Hip fractures will continue to be a major healthcare concern going forward due to our ageing population. As our study has excluded NSA as an independent risk factor for timing to second hip fracture, further studies may now be carried out to look for other potentially modifiable risk factors for predicting timing to second hip fracture.

## Ethical approval

This study received ethical approval from the SJH/TUH Joint Research Committee under protocol REC: 2020–10.

## Competing interests

The authors declare no competing interests.

## Data Availability

No datasets were generated or analysed during the current study.
